# Mendelian Randomization Study on Causal Association of Pyroglutamine with COVID-19

**DOI:** 10.1007/s44197-022-00073-1

**Published:** 2022-10-11

**Authors:** Wenting Su, Shan Zhou, Gaizhi Zhu, Yaqi Xu, Ran Gao, Min Zhang, Qi Zeng, Renxi Wang

**Affiliations:** Beijing Institute of Brain Disorders, Laboratory of Brain Disorders, Ministry of Science and Technology, Collaborative Innovation Center for Brain Disorders, Capital Medical University, No. 10 Xitoutiao, You An Men, Beijing, 100069 China

**Keywords:** Pyroglutamine, Genetic variants, COVID-19, Genome-wide association study, Mendelian randomization

## Abstract

**Background:**

Glutamine family amino acids such as glutamate, pyroglutamate, and glutamine have been shown to play important roles in COVID-19. However, it is still unclear about the role of pyroglutamate in COVID-19. Thus, we use a two-sample Mendelian randomization (MR) study to identify the genetic causal link between blood pyroglutamine levels and COVID-19 risk.

**Methods:**

Pyroglutamine genetic instrumental variables (IVs) were chosen from the largest pyroglutamine-associated genome-wide association studies (GWAS). The largest COVID-19 GWAS dataset was employed to evaluate the causal link between blood pyroglutamine levels and COVID-19 risk using two-sample MR analysis.

**Results:**

We found no significant pleiotropy or heterogeneity of pyroglutamine-associated genetic IVs in COVID-19 GWAS. Interestingly, we found that as pyroglutamine genetically increased, the risk of COVID-19 decreased using inverse variance weighted (IVW) (Beta = − 0.644, *p* = 0.003; OR = 0.525, 95% CI [0.346–0.798]) and weighted median (Beta = − 0.609, *p* = 0.013; OR = 0.544, 95% CI [0.337–0.878]).

**Conclusion:**

Our analysis suggests a causal link between genetically increased pyroglutamine and reduced risk of COVID-19. Thus, pyroglutamine may be a protective factor for patients with COVID-19.

## Introduction

In 2019, severe acute respiratory syndrome coronavirus 2 (SARS-CoV-2) has caused humans mild to severe acute respiratory syndrome called as corona virus disease (COVID-19) [1]. The ongoing COVID-19 pandemic have affected millions of people and caused a serious public health threat worldwide [2]. To effective control of its spread, it is important to understand the risk and protective factors involved in this disease.

The major COVID-19-associated metabolic risks could predispose certain groups to severe COVID-19 complications [3]. Compare with healthy controls, COVID-19 and COVID-19-like patients have enriched serum L-glutamic acids [4]. L-pyroglutamic acids, a cyclized derivative of L-glutamic acids, have been proposed as potential antiviral candidates to treatment of COVID-19 based on calculations and molecular docking [5]. Deficiency of glutamine, made via the action of glutamine synthetase from glutamate and ammonia, has been proposed to be the central metabolic characteristic of COVID-19 and its high-risk groups [3]. The usage of glutamine supplementation has been suggested to prevent the development of severe COVID-19 pneumonia [3]. However, it is still unclear about the role of pyroglutamate, a cyclic amino acid formed as a result of dehydration of glutamate, in COVID-19.

Many factors including reverse causation and confounding bias observational studies and result in the absence of high-quality randomized controlled trials (RCTs) [6–14]. Mendelian randomization (MR) studies highly similar to RCTs, using genetic variants independent of many factors that bias observational studies such as RCTs, have many advantages over RCTs in assessing the causal link between an exposure and an outcome [8–14]. Thus, pyroglutamine genetic variants were used as instrumental variables (IVs) to identify the causal association of pyroglutamine with COVID-19 in two-sample MR analysis.

## Materials and Methods

### Pyroglutamine Genetic Instrumental Variables (IVs)

Pyroglutamine genetic IVs were chosen from the largest pyroglutamine GWAS. This GWAS was reported by So-Youn Shin et al. in 2014 [15]. Its primary aim is to provide unprecedented insights into how genetic variation influences metabolism and complex disease using genome-wide association scans with high-throughput metabolic profiling. Pyroglutamine GWAS has 7800 European participants. The summary statistics for genetic associations of pyroglutamine are available at https://gwas.mrcieu.ac.uk/datasets/met-a-501. Four independent pyroglutamine genetic IVs were obtained based on the following three criteria: (1) genome-wide significance threshold *p* value < 5 × 10^−8^; (2) *r*^2^ < 0.001, indicating no linkage disequilibrium between SNPs by the Linkage disequilibrium (LD) analysis using LDlink (https://ldlink.nci.nih.gov/?tab=ldmatrix, CEU); (3) no effects on other potential risk factors including body mass index, smoking, and blood pressure. Detailed information about these IVs is shown in Table 1. *R*^2^ is the proportion of pyroglutamine variance explained for each independent SNP and estimated based on beta, standard error, and sample size. *F*-statistic was used to assess the strength of relationship between IVs and phenotype, and calculated using the following equation: *F* = *R*^2^ * (*N*-2)/(1−*R*^2^) [16], where *R*^*2*^ is the proportion of pyroglutamine variance, *k* is the number of instruments used in the model and *n* is the sample size.

**Table 1:** Pyroglutamine genetic instrumental variables (IVs)

SNP	EA	NEA	EAF	Beta	SE	*p* val	Sample size	*R*^2 (%)^	*F*-statistics
rs715	C	T	0.286	− 0.036	0.004	2.46E-16	7354	0.91	67.31
rs17279437	A	G	0.095	0.059	0.006	1.25E-20	7354	1.18	87.70
rs11613331	A	G	0.553	0.037	0.004	2.23E-25	7354	1.45	107.93
rs1600760	A	T	0.663	− 0.022	0.004	5.12E-09	7354	0.46	34.08

*R*^2^ the proportion of pyroglutamine variance explained by the selected genetic variants, *F*-statistics the strength of relationship between IVs and phenotype

*IVs* instrumental variables, *SNP* single-nucleotide polymorphism, *EA* effect allele, *NEA* non-effect allele, *EAF* effect allele frequency, *Beta* the regression coefficient based on the pyroglutamine effect allele, *SE* standard error

### COVID-19 GWAS Dataset

The largest GWAS for COVID-19 (RELEASE 4) was described by the COVID-19 Host Genetics Initiative in 2020 [17]. This GWAS dataset is based on 14,134 cases and 1,284,876 controls with European ancestry. The profile of this GWAS is provided in Table 2. The summary dataset is available at https://gwas.mrcieu.ac.uk/datasets/ebi-a-GCST010780.

**Table 2:** Corona virus disease 2019 (COVID-19) genome-wide association study (GWAS)

GWAS ID	Year	Trait	ncase	ncontrol	nsnp	Population	PMID
ebi-a-GCST010780	2020	COVID-19 (RELEASE 4)	14,134	1,284,876	12,508,741	European	32404885

*COVID-19* corona virus disease 2019, *GWAS* genome-wide association study, *GWAS ID* GWAS identity, *ncase* the number of COVID-19 case, *ncontrol* the number of the control, *nsnp* the number of single-nucleotide polymorphism, *PMID* pubMed unique identifier

### Association of Pyroglutamine Genetic Instrumental Variables (IVs) in COVID-19 GWAS

Potential proxy SNPs were identified by the LD proxy Tool (*r*^2^ > 0.8) when pyroglutamine IVs could not be found in COVID-19 summary statistics. However, we were able to successfully extract four independent pyroglutamine genetic IVs from the COVID-19 GWAS summary dataset. The association of four independent pyroglutamine genetic IVs with COVID-19 GWAS is shown in Table 3.

**Table 3:** Association of pyroglutamine genetic instrumental variables (IVs) with COVID-19 GWAS

SNP	Exposure (pyroglutamine) GWAS			Outcome (COVID-19) GWAS		
	Beta	SE	*p* val	Beta	SE	*p* val
rs11613331	0.037	0.004	2.23E-25	− 0.024	0.013	0.055
rs1600760	− 0.022	0.004	5.12E-09	− 0.002	0.013	0.891
rs17279437	0.059	0.006	1.25E-20	− 0.067	0.023	0.004
rs715	− 0.036	0.004	2.46E-16	0.015	0.014	0.289

*IVs* instrumental variables, *COVID-19* corona virus disease 2019, *GWA*S genome-wide association study, *SNP* single-nucleotide polymorphism, *Beta* the regression coefficient based on pyroglutamine raising effect allele, *SE* standard error

### Pleiotropy and Heterogeneity Test

MR-egger_intercept and MR-pleiotropy residual sum and outlier (MR-PRESSO) tests have previously been described to test the pleiotropy [18]. MR_Egger is based on the same regression model with inverse variance weighted (IVW), but allows and accounts for the potential pleiotropy using the MR-Egger intercept test [18–20]. If the selected genetic variants are not pleiotropic, then the MR_Egger intercept term should tend to zero as the sample size increases [20]. MR-PRESSO could detect and correct for the horizontal pleiotropy via outlier removal (the MR-PRESSO outlier test) [18]. MR-egger_intercept and PRESSO methods were used to test the pleiotropy of independent pyroglutamine genetic IVs in COVID-19 GWAS dataset. MR_egger and IVW in Cochran’s *Q* statistic have been broadly used to examine the heterogeneity [21, 22]. Cochran’s *Q* statistic could provide evidence of heterogeneity due to pleiotropy or other causes.[21]. Thus, MR egger and IVW in Cochran’s *Q* statistic were used to test the heterogeneity of independent pyroglutamine genetic IVs in COVID-19 GWAS dataset. Table 4 demonstrates the results about pleiotropy and heterogeneity test. When *p* > 0.05, there is no significant pleiotropy or heterogeneity of four independent pyroglutamine genetic IVs in COVID-19 GWAS.

**Table 4:** Pleiotropy and heterogeneity test of pyroglutamine genetic instrumental variables (IVs) in COVID-19 GWAS

Pleiotropy test				Heterogeneity test					
MR_Egger			Presso	MR Egger			IVW		
Intercept	SE	*p* val	*p* val	*Q*	*Q*_df	*Q_p* val	*Q*	*Q*_df	*Q_p* val
0.043	0.025	0.224	0.454	0.266	2	0.876	3.285	3	0.350

*p* val > 0.05 represent no significant pleiotropy. *Q_p* val > 0.05 represents no significant heterogeneity

*IVs* instrumental variables, *COVID-19* corona virus disease 2019, *GWAS* genome-wide association study, *IVW* inverse variance weighted, SE standard error

### MR Analysis

Two methods including IVW and weighted median were used to analyze the causal association of blood pyroglutamine levels with COVID-19. The IVW was selected as the main MR analysis method to combine the variant-specific Wald estimators by taking the inverse of their approximate variances as the corresponding weights [20]. In addition, we also selected the weighted median that could produce consistent estimates even up to 50% of selected genetic variants are not valid [18–20]. Table 5 demonstrates the results about MR analysis. When *p* < 0.05, there is the causal association of pyroglutamine with COVID-19.

**Table 5:** The causal association of blood pyroglutamine levels with COVID-19

Method	nsnp	Beta	SE	*p* val	OR	OR_lci95	OR_uci95
IVW	4	− 0.644	0.213	0.003	0.525	0.346	0.798
Weighted median	4	− 0.609	0.244	0.013	0.544	0.337	0.878

*COVID-19* corona virus disease 2019, *IVW*, inverse variance weighted, *nsnp* the number of single-nucleotide polymorphism, *Beta* the regression coefficient based on pyroglutamine raising effect allele, *SE* standard error, *p* < 0.05 represents the causal association of the increased pyroglutamine levels with COVID-19, *OR* odds ratio, *OR_lci95* lower limit of 95% confidence interval for OR, *OR_uci95* upper limit of 95% confidence interval for OR

### Each SNP Effect Analysis

**Fig. 1:**
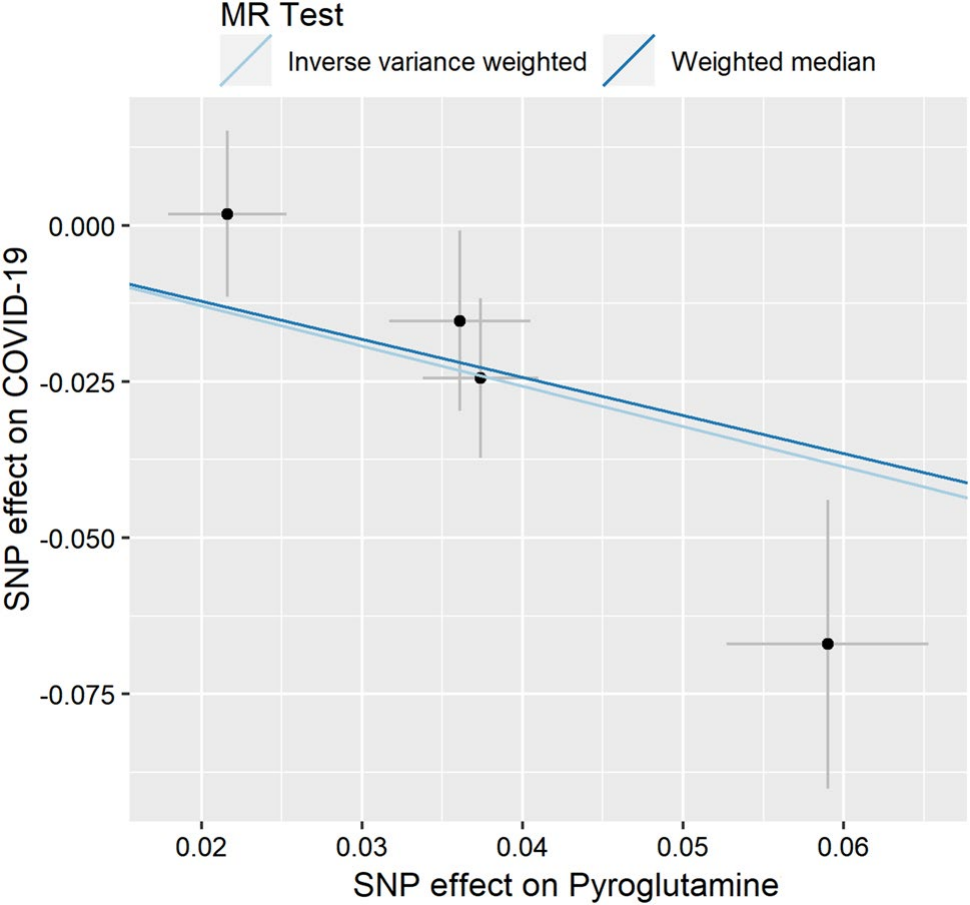
Individual estimates about the causal effect of pyroglutamine on COVID-19. The *x*-axis shows the SNP (single-nucleotide polymorphism) effect and SE (standard error) on each of pyroglutamine. The *y*-axis shows the SNP effect and SE on COVID-19. The regression line for inverse variance weighted (IVW) and weighted median is shown

**Fig. 2:**
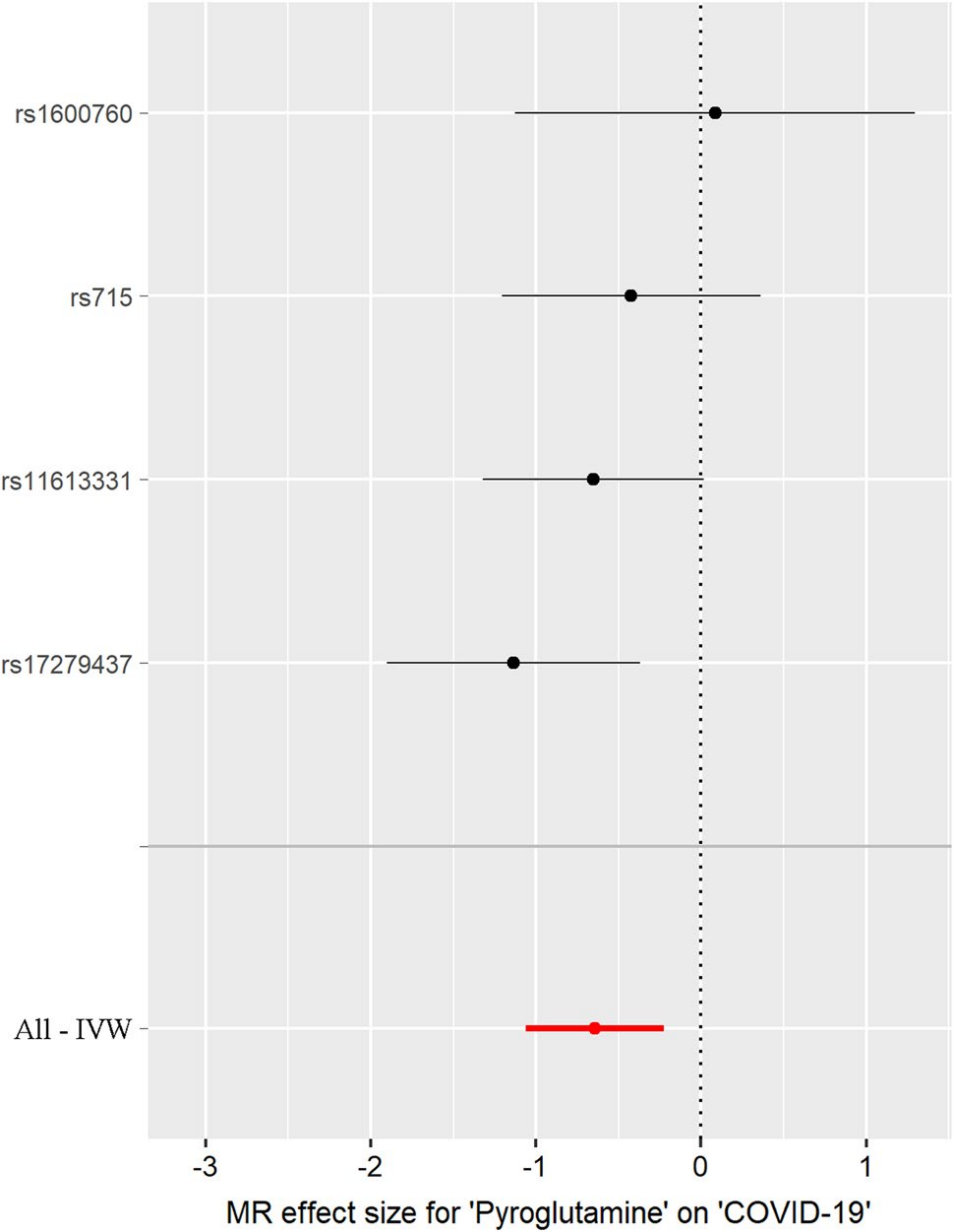
Forest plot of pyroglutamine-associated with risk of COVID-19. The *x*-axis shows MR effect size for pyroglutamine on COVID-19. The *y*-axis shows the analysis for each of SNPs

**Fig. 3:**
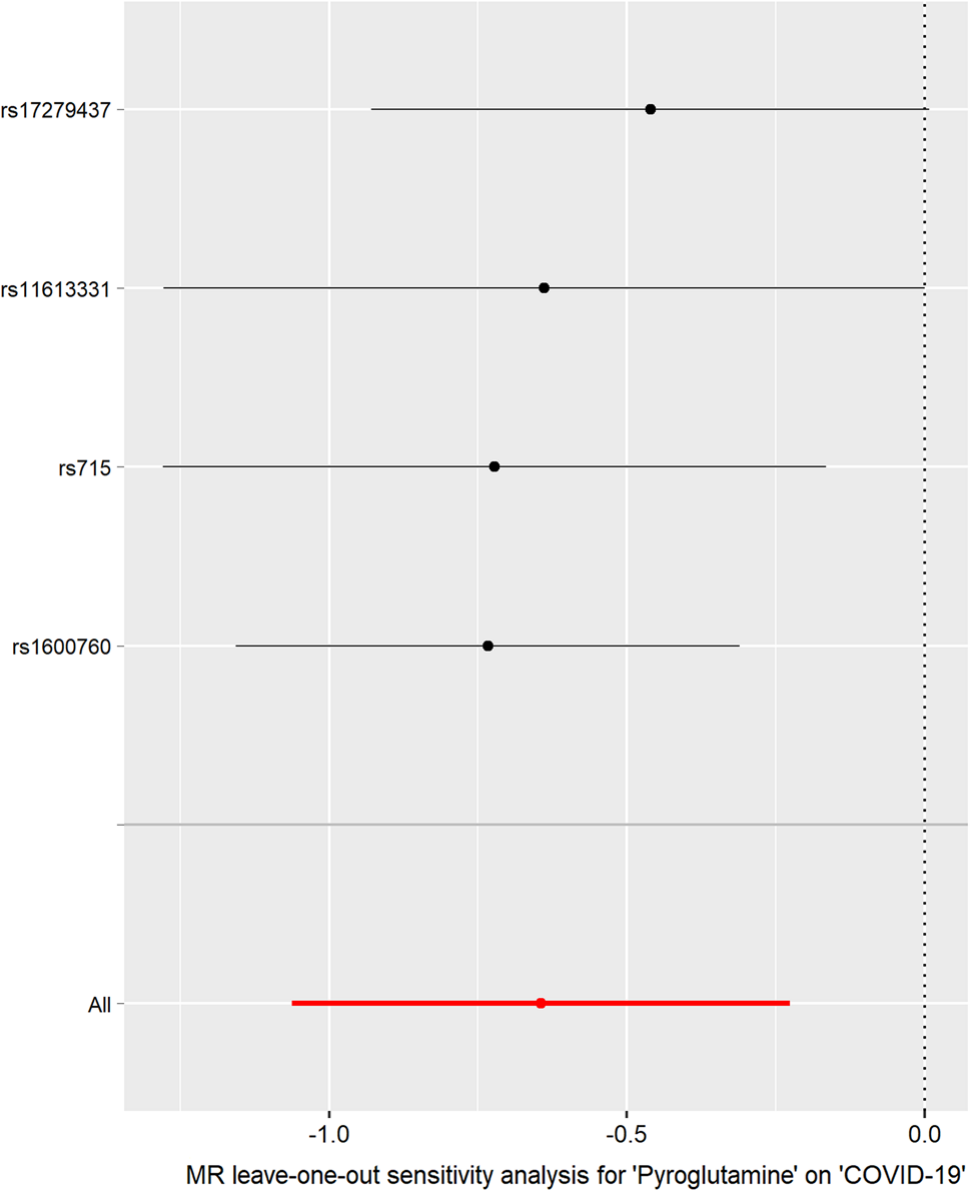
MR leave-one-out sensitivity analysis for the effect of pyroglutamine SNPs on COVID-19. The *x*-axis shows MR leave-one-out sensitivity analysis for pyroglutamine on COVID-19. The *y*-axis shows the analysis for the effect of leave-one-out of SNPs on COVID-19

To analyze each SNP effect, three analysis methods including individual causal effect, each SNP effect size, and SNPs leave-one-out effect were used to explore the effect of pyroglutamine SNP on COVID-19 and shown in Figs. 1, 2, and 3, respectively.

## Results

### Four Pyroglutamine Genetic Instrumental Variables (IVs) Have no Significant Pleiotropy or Heterogeneity

To identify the causal association of pyroglutamine with COVID-19, we first chose four independent pyroglutamine genetic variants as potential IVs from pyroglutamine GWAS (Table 1). All these selected four genetic variants could explain 3.99% variance of blood pyroglutamine levels (Table 1). The *F*-statistics of the selected four IVs were all above the threshold of weak instruments of *F*-statistic < 10 [23], indicating strong IVs for this MR study (Table 1).

We successfully extracted four independent pyroglutamine genetic variants (Table 1) from COVID-19 GWAS dataset (Table 2). The association of pyroglutamine genetic variants within COVID-19 GWAS dataset is shown (Table 3). We found no significant pleiotropy or heterogeneity of four independent pyroglutamine genetic variants in COVID-19 GWAS dataset (Table 4). Therefore, all selected pyroglutamine genetic variants can be taken as the effective IVs in our MR study to explore the causal association of pyroglutamine with COVID-19.

### Pyroglutamine Genetically Reduces COVID-19 Risk

Interestingly, we found that as pyroglutamine genetically increased, the risk of COVID-19 decreased using IVW (Beta = − 0.644, *p* = 0.003; OR = 0.525, 95% CI [0.346–0.798]) and weighted median (Beta = − 0.609, *p* = 0.013; OR = 0.544, 95% CI [0.337–0.878]) (Table 5). Collectively, our data suggested a causal association of genetically increased pyroglutamine levels with the reduced risk of COVID-19.

### Single SNP Effect of Pyroglutamine on COVID-19 is Robust Without Obvious Bias

The individual MR estimates demonstrated that as the effect of each SNP on pyroglutamine increased, the suppressive effect of each SNP on COVID-19 increased using IVW and weighted median (Fig. 1). All effect size analyses suggest that each effect of pyroglutamine SNPs on COVID-19 was robust (Fig. 2). MR leave-one-out sensitivity analysis suggested that removing a specific SNP of the four pyroglutamine SNPs did not change the results (Fig. 3). Altogether, these results indicate that our data were robust without obvious bias.

## Discussion

Previous studies have shown that glutamine family amino acids such as glutamate, pyroglutamate, glutamine have important roles in COVID-19 [3–5, 24–26]. In the present study, we used two-sample MR study and found a causal link between genetically increased pyroglutamine levels and reduced risk of COVID-19. Our findings showed that genetic predisposition to a higher pyroglutamine level may be genetically associated with lower risk of COVID-19.

Serum pyroglutamine has been reported to be inversely associated with overall prostate cancer (OR = 0.53, 95% CI [0.36–0.78], *p* = 0.0013) [25]. The metabolomics profiles of severe COVID-19 patients and patients with advanced cancer are similar and SARS-CoV-2 infection promotes a cancer-like metabolism [3]. These researches propose that pyroglutamine may be inversely associated with COVID-19. As expected, our results proved the proposal.

The concentrations of pyroglutamine in the blood serum were increased in patients who took antihypertensives such as beta-blockers [26]. Hypertension has emerged as significant risk factors for COVID-19 [27]. Antihypertensive drugs have been implicated in COVID-19 susceptibility and severity [28]. These studies suggest that pyroglutamine may be positively associated with anti-COVID-19 therapy.

Pyroglutamine is a cyclic derivative of glutamine related to pyroglutamic acid [29]. Pyroglutamate (or pyroglutamic acid) is an intermediate in the glutathione metabolism and a marker of glutathione deficiency [30]. Glutathione is one of the most potent anti-oxidants in the human body. In fact, glutathione plays an important role in cell proliferation [31]. An increase of intracellular oxidative stress likely leads to its cytotoxicity, inhibition of cell proliferation, and induction of cell death [32]. It also suggests that the glutamic acid rather than the cysteine released from glutathione is responsible for the cell proliferation [33]. The virus-host-specific interactions, molecular targets on host cell deaths, and the involved signaling are crucial issues, which become potential targets for treatment [34]. Anti-COVID-19 action of puerarin was associated with the suppression of oxidative stress and inflammatory cascades, and cell apoptosis [35]. Thus, it is possible that pyroglutamine suppresses oxidative stress to reduce host cell apoptosis in patients with COVID-19.

This study has several strengths. First, pyroglutamine genetic IVs are chosen from the largest pyroglutamine GWAS reported by So-Youn Shin et al. in 2014 [15]. Second, we used the largest GWAS for COVID-19 described by the COVID-19 Host Genetics Initiative in 2020 [17]. Third, both pyroglutamine genetic IVs and COVID-19 GWAS are from European ancestry. Thus, it removed the influence of population stratification. Fourth, four independent pyroglutamine genetic IVs were successfully extracted from COVID-19 GWAS. Fifth, we used four different analysis methods demonstrated no significant pleiotropy or heterogeneity of pyroglutamine genetic IVs as the effective IVs. Sixth, two MR analysis including IVW and weighted median proved the causal link between genetically increased pyroglutamine levels and reduced risk of COVID-19. Finally, all three methods demonstrated that each effect of pyroglutamine SNPs on COVID-19 was robust without obvious bias.

This study has several limitations. First, because pyroglutamine genetic IVs and COVID-19 GWAS are from European ancestry, similar results in other ancestries need be proven. Second, it is necessary to clarify whether pyroglutamine could reduce the risk of COVID-19 by randomized controlled trials. Third, it is still unclear about the underlying mechanism by which pyroglutamine genetically reduced COVID-19 risk that is worth to be explored in the future. Finally, further research on pyroglutamine is needed since too little is known so far about its physiological role [26].

In conclusion, our analysis suggested a causal link between genetically increased pyroglutamine and reduced risk of COVID-19. Thus, pyroglutamine may be a protective factor for patients with COVID-19.

## Data Availability

The datasets generated during and/or analyzed during the current study are available from the corresponding author [Renxi Wang], on reasonable request.

## Abbreviations

IVs: Instrumental variants
SARS-CoV-2: Severe acute respiratory syndrome coronavirus 2
COVID-19: Corona virus disease 2019
GWAS: Genome-wide association study
MR: Mendelian randomization
SNP: Single-nucleotide polymorphism
IVW: Inverse variance weighted
